# Hepatitis C Virus Assembly Imaging

**DOI:** 10.3390/v3112238

**Published:** 2011-11-15

**Authors:** Costin-Ioan Popescu, Yves Rouillé, Jean Dubuisson

**Affiliations:** 1 Institute of Biochemistry, The Romanian Academy, Splaiul Independentei 296, 060031 Bucharest 17, Romania; 2 Inserm U1019, CNRS UMR8204, Center for Infection and Immunity of Lille (CIIL), Institut Pasteur de Lille, Université Lille Nord de France, Lille 59021, France; E-Mails: yves.rouille@ibl.fr (Y.R.); jean.dubuisson@ibl.fr (J.D.)

**Keywords:** hepatitis C virus, virus assembly, cellular imaging

## Abstract

Hepatitis C Virus (HCV) assembly process is the least understood step in the virus life cycle. The functional data revealed by forward and reverse genetics indicated that both structural and non-structural proteins are involved in the assembly process. Using confocal and electron microscopy different groups determined the subcellular localization of different viral proteins and they identified the lipid droplets (LDs) as the potential viral assembly site. Here, we aim to review the mechanisms that govern the viral proteins recruitment to LDs and discuss the current model of HCV assembly process. Based on previous examples, this review will also discuss advanced imaging techniques as potential means to extend our present knowledge of HCV assembly process.

## 1. Introduction

Hepatitis C virus (HCV) represents a global health problem with 130 million people infected worldwide [1]. HCV infection is generally chronic and the virus primarily infects human hepatocytes, which over time leads to chronic inflammation of the liver, progressive fibrosis and development of hepatocellular carcinoma.

**Figure 1.**
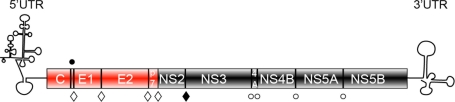
Representation of the genetic organization of hepatitis C virus (HCV). The N-terminal part of the genome comprises the structural elements core (C)—the forming unit of the capsid and the envelope glycoproteins, E1 and E2. The C-terminal part of the genome consists of the non-structural proteins (NS3 to NS5B) responsible for HCV genome amplification. p7 and NS2 are non-structural protein dispensable for replication, but essential for viral assembly. HCV proteins are synthesized as a polyprotein, which is processed by endogenous proteases (full circle—signal peptide peptidase, open diamond— signal peptidase) or viral encoded proteases: NS2 (full diamond) and NS3 (open circle) to generate 10 mature proteins.

HCV is a positive stranded RNA virus, which belongs to the *Flaviviridae* family [2]. Upon entry in the hepatocyte, the viral genome is translated into a polyprotein, which is sequentially cleaved by both endogenous and viral peptidases to produce 10 mature proteins (Figure 1). The N-terminus of the polyprotein consists of the structural proteins: the core protein and the envelope glycoproteins, E1 and E2. The C-terminal part comprises the non-structural proteins NS3 to NS5B, which are the viral components of the replication complex. Between the structural and non-structural proteins there are two additional proteins most likely non-structural, called p7 and NS2. They are dispensable for replication, but they are emerging as major players in HCV assembly.

For reasons that remain partly unknown, serum-derived HCV cannot be propagated in cell culture. Therefore HCV research has largely depended on surrogate systems. For instance, replicon systems were developed to investigate HCV genome replication [3], and replication-deficient retroviruses pseudotyped with HCV envelope glycoproteins (HCVpp) were engineered to study HCV entry [4–6]. Unfortunately, no convincing surrogate model could be developed to study HCV assembly, and for this reason, HCV morphogenesis is the least understood step in the life cycle of this virus. Nevertheless, in 2005, several teams finally succeeded in propagating HCV in cell culture (HCVcc) [7–9]. Since then, data have been accumulating regarding the assembly events. The peculiarity of HCV and the other members of the *Flaviviridae* family in general is the crucial role played by both structural and non-structural proteins in the assembly process. Indeed, genetic, cell biology and biochemistry data highlight the role of practically all HCV proteins in the assembly process (reviewed in [10]). Another major specific feature of HCV is its strong dependence on lipoprotein biogenesis for its morphogenesis (reviewer in [11]). Despite the progress made on HCV assembly during the last six years, there is still no unified picture of the assembly process. In order to reach this goal, different groups have been using electron and fluorescence microscopy trying to visualize the viral particle and to get insight into the scenario of the assembly process, respectively. This review aims to summarize the achieved progress and to suggest a working model and different perspectives.

## 2. HCV Protein Components

The core protein and the envelope glycoproteins are the known viral protein components of HCV particle (Figure 1). Core is a dimeric protein [12] with two domains: a charged hydrophilic domain (D1) supposed to interact with the viral RNA during nucleocapsid formation and a hydrophobic domain (D2) which binds endoplasmic reticulum (ER) outer leaflet [13]. The envelope glycoproteins, E1 and E2, are the protein components of the viral envelope (reviewed in [14]). They are type I transmembrane glycoproteins with up to five and eleven glycans, respectively. E1 and E2 form a non-covalent heterodimer, which is believed to be the basic functional unit of the viral envelope [15]. These proteins play a major role during virus entry into the hepatocyte and their entry functions are tightly dependent on proper assembly of the viral particle. Recently, E1E2 heterodimers were shown to form large complexes stabilized by intermolecular disulfide bridges on the surface of the viral particle [16]. These covalent interactions likely play a role in the spatiotemporal control of the entry process.

The p7 and NS2 polypeptides are non-structural proteins dispensable for genomic replication. p7 is a small viroporin with ion channel properties (reviewed in [17]). It is involved in the assembly and release of the viral particle both in cell culture and in *in vivo* models [18–20]. NS2 is a multispanning transmembrane protein with multiple functions. It has autoprotease activity catalyzing the processing of its C-terminus. Recently, it was also reported to be central for the assembly process by connecting the structural and non-structural proteins during virus assembly (reviewed in [10]).

The non-structural proteins NS3 to NS5B are the viral components of the replication complex (Figure 1). The NS3 protein possesses two domains: a serine protease and a helicase. NS3 protease domain forms a complex with NS4A, a small hydrophobic polypeptide, which serves as a cofactor. This protease domain processes the downstream cleavage sites of the polyprotein. This processing is essential for viral replication, and as a consequence it is the most advanced drug target for anti-HCV therapy [21]. Beside its crucial role in replication, NS3 was also shown to be involved in the assembly process [22]. NS4B is a hydrophobic protein, which has four transmembrane passages and it induces specific changes in the membranous architecture, called “membranous web” [23]. The membranous web is essential for the efficacy of the viral replication [24]. NS4B seems also to participate in the assembly process [25]. NS5A is a multifunctional protein, which is involved in both replication and virion assembly [26–28]. Besides its N-terminal amphipathic region that enables the protein to bind to phospholipid monolayers, NS5A has three domains with different roles. Domain I is well structured and is involved in replication [29], whereas domains II and III are natively unfolded with domain III playing a role in virion assembly [26,28,30–32]. NS5A has two phosphorylation states (basal and hyperphophorylated), which affect the assembly process and regulate viral RNA replication [28,33]. NS5B, being an RNA-dependent RNA polymerase, is the central component of the replication complex. NS5B is associated to ER membrane by a C-terminal transmembrane domain (reviewed in [34]). NS5B replicates the viral genome with the help of host specific factors and the other viral proteins of the replication complex.

As mentioned above, both structural and non-structural proteins take part in the assembly process. Thus, the particle formation should be a tightly synchronized event in order to switch from replication to assembly. Ideally, to completely understand the process, we should visualize it. Hence, we will present the progress made in the imaging of each viral/endogenous element involved in HCV morphogenesis and how they crosstalk topologically and functionally towards the assembly event. We will then discuss the relevance of the models and techniques used to the understanding of HCV particle formation. Finally, we will suggest some hypotheses and new approaches to validate them.

## 3. Core and Lipid Droplets in HCV Assembly

**Figure 2.**
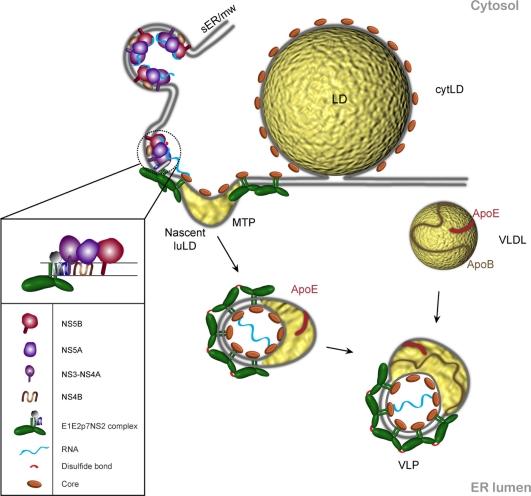
Working model of HCV assembly. Viral assembly is triggered by the encounter of three modules: core, E1E2p7NS2 complex and the replication complex (RC). The assembly site is supposed to be in the membranous microenvironment of the lipid droplet (LD) and the endoplasmic reticulum (ER). The driving force of viral budding potentially comes from three directions: pushing force of the nascent nucleocaspsid, the pulling force of envelope proteins which might stabilize the viral surface architecture by intermolecular disulfide bridges and the force of the nascent luminal LD (luLD) between the ER leaflets. The result is a hybrid lipoviroparticle, which acquires ApoE presumably by its lipid component and in primary hepatocytes the lipoprotein moiety may mature into a VLDL-like structure (adapted from [46]). MTP stands for microsomal triglyceride transfer protein that is involved in the VLDL pathway. sER/mw is for smooth ER/ membranous web where the HCV replication is believed to take place.

As expected, core protein is central to the assembly event as the constitutive unit of the nucleocapsid [35]. It is an α-helical protein which oligomerizes *in vitro* through its basic charged D1 domain (reviewed in [2]). D2 domain provides core lipid binding properties anchoring the protein in the outer leaflet of the ER [13]. By confocal fluorescence microscopy, it was shown that besides its ER localization, core decorates the surface of lipid droplets (LDs) [36,37]. LD is a dynamic organelle, which stores lipids as triacylgricerides (TAGs) and cholesteryl esters surrounded by a phospholipid monolayer. LD surface holds a complex proteome with different functions [38]. LDs are assumed to arise from TAGs accumulation between the two ER leaflets followed by detachment into the cytosol (cytLD) or ER lumen (luLD) [39] (Figure 2). As D2 domain enables core to float in the outer leaflet of the ER, the determinants of core-LD association reside in the same domain [40]. Thus, nuclear magnetic resonance studies showed that D2 consists of two amphiphatic helices separated by a hydrophobic loop. Confocal microscopy analyses with fluorescent reporters identified crucial residues for core-LD association in both structural components of D2 [13]. A second determinant of core-LD association is the processing of the core C-terminus by a signal peptide peptidase [40]. Importantly, the subcellular localization of core around the LDs was confirmed in the HCVcc system [41–43]. By 3D reconstruction of confocal images taken during infection, it was shown that core is gradually decorating the surface of the LDs [41]. The extent of LD core coverage correlated with the production of infectious particles.

In addition to being located around LDs, core protein also induces their redistribution from a scattered distribution within the cytoplasm to a perinuclear region [44]. Viral RNA is replicated in special perinuclear membranes called membranous webs [24], and the LD redistribution might create the lipid microenvironment where the structural and non-structural elements involved in assembly are most likely to interact [42]. Adipose differentiation-related protein (ADRP) is a major protein associated with LDs in the hepatocyte [45]. Interestingly, core protein dislocates ADRP from the surface of LDs, determining its microtubule dependent movement towards the microtubule organizing centre [44]. This model assumes that LD redistribution enhances the encounter between the core and the replication complexes during the assembly event [44]. However, core redistribution around the nucleus could also be explained by the LD regression/regeneration model. It suggests that cytoplasmic LDs regress at ER membranes and regenerate at distant domains rich in core protein, which replaces the ADRP on the surface of LDs, generating their formation and aggregation around the nucleus [45]. In both cases, the core-LD association is directly proportional to the extent of LD redistribution in the perinuclear region.

Interestingly, when comparing viruses with different assembly efficacies, it was found that the core-LD extent of association is inversely proportional to virion production [47]. Moreover, the main determinant of the different assembly efficacies was identified in the D2 of core protein. Indeed, a single mutation in this domain was shown to increase virion production, whereas it reduced its distribution around the LDs [47]. If one considers the microtubule redistribution model, the more efficiently the core protein on the incoming LDs is used in the assembly process, the faster the LD returns to its cytosolic localization probably by ADRP recoating. We should expect that the more mobile the core protein, the better it will partition between LDs and the assembly site. However, fluorescence recovery after photobleaching (FRAP) analyses indicate that D2 domain mobility is inversely proportional to virion production [47]. The authors suggest that higher rigidity of the core protein can increase the chances for the protein to interact with the other viral elements during the assembly process (e.g., viral RNA, NS5A). Another titer-enhancing mutation in core protein of the JFH1 isolate is the double substitution F172C, P173S near the C-terminus of the mature protein [48]. The palmitoylation of C172 could be the determinant for this more efficient assembly [49]. Interestingly, this post-translational modification also correlates with an increased partitioning of core to smooth ER membranes associated with LDs, and lower coverage of the LD surface.

On the other hand, when the core-LD association is totally prevented, virus assembly is drastically impaired. Indeed, infectivity, viral RNA and core release are seriously inhibited in these conditions [41,42]. Importantly, there is a close association between recruitment of other viral proteins to LDs and the core-LD association. Indeed, NS2, NS3, NS4AB, NS5A, NS5B localize around the LDs in infected cells [42,44,50–52]. Electron microscopy images showed that while core is tightly associated to LD, NS2, NS5A, E2 and probably E1 and other non-structural proteins also colocalize at close proximity of the LDs [42,52]. Furthermore, *in situ* hybridization analyses indicate that plus and minus strand viral RNAs are also found at the proximity of LDs [42]. In contrast, viruses containing defective mutations in core-LD association do not show accumulation of the other viral elements around the LDs [42,52]. The partition of core between LD and ER membranes and the consequent presence of other viral proteins around the LDs is therefore central to the assembly process.

It is important to note that other proteins can influence the localization of core. Indeed, it was shown recently that p7 and NS2 induce the redistribution of core from LD to ER membrane, paralleling the increase of the viral titers and subsequently assembly efficacy [53]. The channel activity of p7 does not seem to influence this core redistribution. Rather, elements possibly involved in p7-NS2 protein-protein interactions play a role in core subcellular localization [53]. p7 and NS2 have indeed been shown to interact [52], and there is a functional connection between p7 and NS2 in the assembly process as discussed in the next section. Besides the viral proteins involved in assembly, the core-LD association can also be influenced by endogenous proteins. Diacylglycerol acyltransferase-1 (DGAT1), an enzyme involved in LD morphogenesis has recently been demonstrated to interact with core and to enable core-LD association and virus production [54].

## 4. p7 and NS2 Are Central Players in HCV Morphogenesis

**Figure 3.**
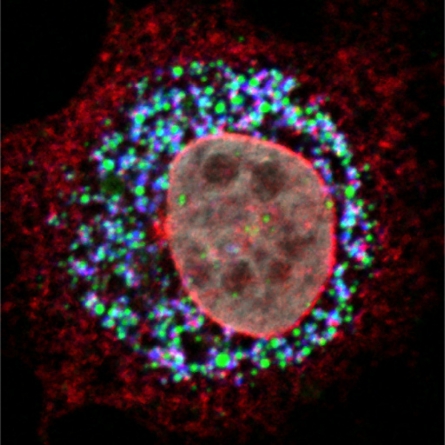
NS2 protein accumulates at the site of virus assembly. Confocal image of an electroporated hepatocyte with the HCV genome shows the accumulation of NS2 (red) and core protein (blue) in close proximity to cytoplasmic LDs (green), the putative site of HCV assembly. The nucleus was counterstained with DAPI (grey).

The participation of non-structural proteins in virion assembly appears to be the paradigm for the *Flaviviridae* family (reviewed in [55]). p7 and NS2 are dispensable for viral replication, but they are crucial for virion assembly and release [18,20,56]. An appropriate subcellular localization of these proteins is therefore essential for HCV morphogenesis. In HCV-infected cells, NS2 accumulates in dotted structures, which are derived from the ER membranes and are juxtaposed to LDs [50,52] (Figure 3). By electron microscopy, NS2 was shown to form clusters in the proximity of LDs in a 200 nm range, which is the resolution of the confocal microscopy [52]. These NS2-containing structures have been proposed to be the subcellular sites of HCV assembly. Indeed, NS2 positive dots co-localize with E1 and E2 envelope glycoproteins as well as with NS3 and NS5A non-structural proteins. Furthermore, these dots are juxtaposed to core protein, which decorates the LDs (Figure 3). The co-localization of all these proteins indicates that membranes harboring replication complexes are juxtaposed to the LDs, however they are in a more peripheral position of the LDs as compared to core protein [42]. When core-LD association is prevented, the NS2 dotted structures still form and their number increases significantly [52]. Thus, it seems that the accumulation of the envelope and non-structural proteins at the assembly sites takes place prior to the arrival of core-loaded LDs. The presence of core might trigger the assembly event since in its absence there is an increase in structures enriched in NS2 and envelope proteins. It is worth noting that core interacts directly with NS5A and this might trigger the assembly process [31].

Genetic data suggest that the transmembrane domain of NS2 is a site of interactions with structural and non-structural proteins (reviewed in [10]). In addition, biochemical experiments indicate that NS2 interacts with E1, E2, p7 and NS3 [50,52,57] to form a large hetero-oligomeric complex, E1E2p7NS2NS3 [57]. Hence, it is likely that some of these proteins travel together to the assembly site. It seems to be the case for E2, p7 and NS2 since deletion of p7 or of the transmembrane domain of E2 impairs their interaction with NS2 as well as the formation of assembly sites [52]. Mutagenesis experiments indicate that the transmembrane region of NS2 is a major determinant of its subcellular localization [52]. Together, these data reinforce the model where E1E2 heterodimer, NS2 and p7 form a functional unit, which migrates to NS5A positive membranes, possibly populated by replication complexes.

When NS2 complexes arrive or form at the NS5A positive membranes, a signal should be sent to initiate the assembly process. As discussed in detail in the next section, NS5A phosphorylation at specific sites in domain III is essential for HCV assembly and core-NS5A direct interaction [28,31]. Interestingly, mutation of these phosphorylation sites in NS5A impaired the formation or stabilization of NS2 dotted structures suggesting a functional connection between NS2 and NS5A in the assembly event [52]. Thus, NS5A phosphorylation might initiate the core-NS5A interaction triggering the assembly process, which is characterized by NS2 dotted structures bridging the structural and non-structural protein towards virion formation.

Unfortunately, the data about p7 subcellular localization are missing from HCVcc system due to the lack of reagents. While it was shown that in recombinant systems p7 localizes both in ER and in mitochondria, in the context of the full length HCV replicon the protein had an exclusive ER localization [58,59]. Until p7 is visualized in a functional infectious virus, we can only speculate that p7 would follow at least partially NS2 localization since these two proteins interact and p7 is a crucial determinant of NS2 subcellular localization [50,52,57].

## 5. HCV Envelope Glycoproteins and Viral Morphogenesis

HCV envelope glycoproteins, E1 and E2, are major components of the viral particle. These proteins consist of a large N-terminal ectodomain and a C-terminal transmembrane domain, anchoring each glycoprotein in a lipid bilayer. The transmembrane domains contain ER retention signals [60,61], responsible for their subcellular localization [43]. In infected cells, E1 and E2 form a non-covalent heterodimer, which is believed to be the basic functional unit of the viral envelope, whereas virion-associated envelope glycoproteins form large covalent complexes stabilized by disulfide bridges [16]. The presence of disulfide bridges between HCV envelope glycoproteins suggests that lateral protein-protein interactions assisted by disulfide-bond formation might play an active role in the budding process of HCV particle. Although they are retained in the ER, over time, HCV envelope glycoproteins co-localize with NS2 dotted structures. This indicates that NS2 sub-compartment is a potential virion assembly platform enriched in envelope proteins and non-structural element involved in both replication and assembly (e.g., NS3 and NS5A) [52].

## 6. Non-Structural Proteins of the Replication Complex Are Involved in HCV Assembly

As for other members of the *Flaviviridae* family [55], HCV non-structural proteins of the replication complex are also involved in virus assembly (reviewed in [10]). Viral replication takes place at specific membranous domains called membranous webs [24]. In the context of the subgenomic replicon, it was shown that the replication complex belongs to two populations: large, static structures representing the membranous webs and small replication complexes which travel long distances in a microtubule dependent manner [62].

In agreement with the functional data, NS3, NS4A, NS4B, NS5A and NS5B were found to accumulate in the proximity of the LDs, at the proposed site of viral assembly [42,43]. Core-LD association is crucial for the recruitment of the non-structural elements possibly present in active replication complexes around the LDs [42]. However, it is not clear how the non-structural proteins are recruited in the proximity of the LDs. It has been supposed that core-induced LD redistribution increases the encounter rate between LDs, core and replication complexes [44]. Another possibility is that LDs form in core rich membrane domains proximal to replication complexes [45,63]. In any case there should be a mechanism, which controls the distribution of non-structural proteins between the ER and LDs. NS5A seems to be a crucial factor in the localization of all the replication complex elements, possibly determining the recruitment of active replication complexes at the assembly sites [42]. In line with this scenario, double stranded RNA was found associated with NS5A in the proximity of LDs [64]. NS5A is also believed to be an important connecting element between the replication and assembly steps [26,28,33]. An interesting structural element of NS5A is its amphipathic N-terminus which enables the protein to bind to phospholipid monolayers. It is crucial for the protein localization and function during replication [65,66]. The same element might be also involved in positioning NS5A during the assembly process.

NS5A plays a central role in the recruitment of the replication complexes around the LDs. Indeed, a triple alanine substitution in domain I of NS5A prevents the recruitment of the protein and other non-structural proteins to the LDs [42]. On the other hand, domain I is involved in the mode of action of a class of antiviral compounds. These compounds redistribute the NS5A from punctuated membranous webs to LDs, drastically inhibiting the replication process. Interestingly, the effect is specific to NS5A but not the other non-structural proteins in the replication complex [67]. Thus, domain I may be involved in both preserving the NS5A function in the active replication complexes and mobilizing these complexes towards LDs for assembly to take place.

We have a poor understanding of what happens when the replication complexes encounter the structural elements in the proximity of LDs to trigger the particle assembly. Deletion analyses in domain III of NS5A showed that this domain is crucial for virus assembly [26,28,31]. Deletions in domain III do not affect the subcellular localization of NS5A around the LDs. They rather impair core co-localization with LDs [26,28]. Thus, domain III is involved in a later stage of assembly when the replication complex is already present in the proximity of LDs. It has been suggested that domain III of NS5A might be involved in the recruitment of replication complexes to core vicinity [26]. A direct interaction between core and NS5A has been reported [31]. Furthermore, three phosphorylated serine residues in the C-terminus of domain III are crucial for this interaction as well as for virus production [28,31].

Beside the replication complex proteins, NS5A also influences the subcellular localization of other viral proteins involved in viral assembly like NS2. The dotted structures enriched in envelope proteins juxtaposed to core and LDs are stabilized by the phosphorylation of one serine residue in domain III of NS5A, which is essential for viral assembly [28,52].

## 7. HCVcc Model and Assembly Imaging

Although HCV can now be propagated in cell culture, the amount of viral particles produced remains rather modest as compared to other viruses. In the current experimental conditions, we can roughly estimate that Huh7 cells produce in the range of one infectious HCV particle per cell per infectious cycle. Imaging such a rare event remains therefore very challenging.

At this stage of our knowledge, we can propose that viral assembly occurs in three steps: nucleocapsid formation, budding and maturation to the infectious particle [68]. VLDL pathway is closely related to the infectious particle production. Some reports suggested that both ApoB and MTP are involved in HCV assembly [68,69]. The virion was shown to have a VLDL and LDL like lipid composition and to be associated to ApoE which is essential for the infectious virus assembly [68–71]. It is not clear in which step of viral assembly ApoE is involved. Core secretion is not significantly affected by ApoE [72,73]. Hence, ApoE might be involved either in the budding process or in particle maturation in the ER and a post-ER compartment. The latter step could be more likely to be controlled by ApoE since infectious particle release is dependent on ApoE secretion [72]. It is believed that the lipoviroparticle may fuse further with VLDL in the ER and follow a VLDL like maturation in the secretory pathway. Due to an inefficient VLDL assembly pathway in Huh7 cells, this step is impaired (Figure 2) (reviewed by [46]). Thus, we may conclude that the HCVcc system is suitable for imaging of the first two stages of the assembly process. For a better understanding of the assembly, systems with higher specific infectivity would be required. In principle that could be obtained in primary hepatocytes based systems which are beginning to emerge [74,75].

## 8. Imaging Techniques and HCV Assembly

The majority of the studies of HCV assembly have been performed by confocal microscopy on fixed cells. To answer questions related to the dynamic of the assembly process, viral proteins should be functionally labeled with fluorescent tags in the context of an infectious virus and more advanced techniques like spinning disk and time-lapse confocal microscopy should be used. These would bring valuable insight in the understanding of the connection between LDs and all the viral proteins involved in HCV assembly.

It would be also of great interest to use higher resolution approaches such as stimulated emission depletion (STED) microscopy, stochastic optical reconstruction microscopy (STORM) or photoactivated localization microscopy (PALM), which have enhanced resolution that is up to 10 times higher or better than that of conventional optical microscopy, with lateral resolution to approximately 20 nm and axial resolution to approximately 50 nm, comparing to the classical light microscopy resolution limits of 200 nm and 600 nm, respectively [76]. The use of these techniques could help visualize the encounter of the different modules (Core, E1E2p7NS2, and the replication complex), which cooperate during HCV assembly.

Electron microscopy is still a method of choice for high resolution imaging of the localization of intracellular biomolecules, which often is crucial to understand their function. Traditionally, light and electron microscopy observations are carried out in different samples. This makes it difficult to observe rare events, or events occurring in a limited number of cells in a population, a situation thought to occur in the study of HCV assembly, as discussed earlier. Another limitation in electron microscopy analyses of HCV assembly is the heterogeneity of HCV particles, which makes them difficult to visualize in infected cells [77]. The development of various correlative fluorescence and electron microscopy techniques now allows for high resolution imaging by electron microscopy of the same structure observed by light microscopy. Thus, an event visualized at low resolution by light microscopy or a single cell displaying a phenotype of interest within a population can now also be studied at high resolution by electron microscopy [78]. The use of correlative fluorescence and electron microscopy should help improve our knowledge of the morphology of HCV assembly sites.

Assembly can also be viewed as a series of regulated protein-protein interactions. Deciphering these interactions within the cell will require fluorescence-based techniques capable of measuring these interactions *in vivo*, such as fluorescence resonance energy transfer (FRET) or Fluorescence cross-correlation spectroscopy (FCCS) techniques. FRET has already been used successfully to image HIV virion morphogenesis [79]. Such kinds of assays should provide us with the opportunity of revealing the chronology, dynamics and intracellular distribution of interactions occurring during HCV assembly. However, a major drawback in applying these techniques to HCV assembly has been the great difficulties in inserting fluorescent tags in HCV proteins in the context of an infectious virus. A major step towards this goal has been achieved with the construction of a functional recombinant virus harboring a fluorescently labeled NS5A by adaptation of a full-length EGFP-NS5A replicon [80,81].

## 9. Conclusions

Imaging of HCV infected cells together with biochemical studies are beginning to provide some hints on HCV morphogenesis. Our current working model is depicted in Figure 2. There are three functional modules involved in assembly: core, NS2 complexes containing the envelope proteins, p7 and maybe NS3, and the replication complexes. NS2 complexes reach the replication complexes independently of core protein. Core protein induces LD relocalization/regeneration, enabling the three modules of assembly to encounter in a favorable assembly environment. Viral budding is induced by the pushing force of the nucleocapsid [35] corroborated with the pulling force of envelope proteins [82] and potentially the accumulating neutral lipids between the two leaflets of the ER. The result would be a hybrid lipoviroparticle [83], which matures further by interaction with ApoE. In cells with an efficient VLDL pathway metabolism, the lipoprotein moiety may mature into a VLDL-like structure [46].

Although a picture is now emerging on the assembly process, there are still many unanswered questions. One of the most basic questions concerning HCV assembly is where does it exactly occur? The answer will probably come when we will be able to image the assembly intermediates using high resolution imaging techniques. A second question is how the assembly components are meeting at the assembly sites? This problem concerns the different scenarios of core-LD association and LD intracellular trafficking, the topological and functional connection between the replication complexes, LD and core protein and how they participate alongside the envelope proteins towards the budding process? What is the role of p7 and NS2 in the assembly process? A third question relates to the involvement of lipid metabolism in particle formation/maturation. VLDL pathway is closely related to infectious particle production [68–70]. ApoE was shown to be essential for infectious virus assembly (reviewed in [46]). Is ApoE crucial for nucleocapsid assembly, budding or maturation? Core secretion is not significantly affected by ApoE [72,73]. Hence ApoE might be involved either in the budding process or in particle maturation in the ER and a post-ER compartment. The latter step could be more likely to be controlled by ApoE since viral release is dependent on ApoE secretion [72]. Imaging the relationship between HCV particle and VLDL pathway would be quite difficult due to the major endogenous background of VLDL pathway components. At the end, new cell culture models, advances in viral protein labeling and higher resolution microscopy techniques will contribute to a better understanding of the HCV assembly process, paving the way for new antiviral strategies.
